# Height detection of crop divider toes of sugarcane harvester based on Kalman adaptive adjustment

**DOI:** 10.1038/s41598-023-43952-8

**Published:** 2023-10-05

**Authors:** Chunming Wen, Yunzhi Yan, Wanling Wu, Jianheng Li, Bingxu Hou, Wenxuan Cui, Youzong Huang, Kaihua Li, Xiaozhu Long, Hongliang Nong

**Affiliations:** 1 Guangxi Key Laboratory of Intelligent Unmanned System and Intelligent Equipment, Nanning, China; 2 Guangxi Key Laboratory of Hybrid Computation and IC Design Analysis, Nanning, China; 3 College of Electronic Information, Guangxi Minzu University, Nanning, China; 4 State Key Laboratory for Conservation and Utilization of Subtropical Agro-bioresources, Nanning, China; 5 Nanning Taiyin Technology Co., Ltd, Nanning, China; 6 Guangxi Agricultural Machinery Research Institute, Nanning, China

**Keywords:** Mechanical engineering, Engineering, Electrical and electronic engineering

## Abstract

Crop divider toes are an essential device of sugarcane harvester. Moving forward against the ground is a critical way to improve the harvesting rate of lodged sugarcane. Height detection is the basis for precise control of crop divider toes moving forward against the ground. Due to the current problem of operating difficulties in manually adjusting the height of crop divider, a height detection system based on a millimeter wave radar sensor was designed to detect the height of crop divider toes from the ground. This paper proposed a height detection method of crop divider toes for sugarcane harvester based on Kalman adaptive adjustment. The data measured by the sensor was pretreated to determine whether the height had changed. Reset the Kalman filter and adjust the parameters when changes occur to improve the filter response speed and ranging accuracy. To adapt to the scenario of quickly adjusting the height of crop divider during the traveling process of sugarcane harvester. A one-way ANOVA test and a two-way ANOVA test were conducted on a simulated test platform. The results of the one-way ANOVA test showed that both forward speed and vegetation cover thickness had a significant effect on height detection accuracy. The results of the two-way ANOVA test showed that the interaction of forward speed and vegetation cover thickness did not have a significant effect on ranging accuracy. It was verified through experiments that both the ranging accuracy and the response speed of height change were significantly improved after the processing of the method in this paper. The mean square error after processing was lower than 2.5 cm. The feasibility of the height detection system was verified by field trials. The results of this study will provide a reference for the design of automatic elevation of crop divider.

## Introduction

Sugarcane is the most important sugar crop in China. China’s sugarcane cultivation area ranks third in the world, mainly in Guangxi, Yunnan and Guangdong^1^. In the case of rural labor shortage and the continued sharp rise in labor costs, improving the mechanization of sugarcane harvesting is one of the effective ways to reduce production costs^2^. The use of mechanized production can greatly improve efficiency, but the level of mechanization in the sugarcane harvesting process is not high. Sugarcane growing land in China is dominated by hills and slopes. Due to the annual typhoons and monsoons, sugarcane falls to different degrees during the growing process. Fallen sugarcane makes mechanized harvesting difficult. Crop divider is one of the important devices of sugarcane harvester, the main function is to support the fallen sugarcane in the row, and separate the cross rows of fallen sugarcane. It improves the cutting quality of sugarcane lodging and reduces the loss caused by the cutter’s missed cutting of sugarcane and so on^3^. During mechanized harvesting, if the fallen sugarcane cannot be raised to a certain height, it will cause the cutter to cut the sugarcane multiple times, increasing the rate of root damage^4^. More importantly, this can reduce yields and seriously affect the germination and growth of sugarcane in the next year^5, 6^.

In recent years, to improve the performance of crop divider, many scholars have studied the mechanical structure of crop divider. Most of the sugarcane harvesters on the market today still use spiral crop divider^7^. In field experiments, the effects of different lodging angles, lodging side angles, and sugarcane stem and leaf entanglement on the lifting and lowering performance of crop divider were studied^8^. Gao et al.^9^ developed a mechanical model of sugarcane field growth and analyzed the interaction process between lifting reels and sugarcane. Song et al.^10^ established two-stage spiral sugarcane picking-up machine and applied for the simulation of the virtual prototype, and the structure of the picking-up section was confirmed. Dong et al.^11^ proposed a design method for a new unequal pitch spiral crop divider and verified its feasibility by establishing a physical model through computer simulation. Xie et al.^12^ developed a combined crop divider consisting of a conical spiral drum and a finger chain, and conducted experimental research in the field. Therefore, these studies of crop divider have focused on structural and kinematic parameters, providing a basis for improving the working performance of crop divider. The existing whole stalk harvester cannot automatically adjust the height of crop divider according to changes in the undulation of the ground, and need to adjust the height of crop divider manually. During harvesting, the operator’s view is easily obscured by the sugarcane and dust. Sugarcane harvesting relies heavily on trained operators manually lifting and lowering the crop divider to adapt to uneven terrain. However, it is difficult for the operator to ensure that the crop divider reaches the desired height while balancing driving. Due to the undulating terrain of the sugarcane fields. When the crop divider is far away from the ground, which the front crop divider toes do not fit the ground, the crop divider will not be able to lift the severely fallen sugarcane. Fallen sugarcane that is not lifted for harvesting can be crushed by the harvester tracks and cause damage. Therefore, it is of great significance to strengthen the research on the height adaptive adjustment of crop divider.

The measurement methods for the measuring height of the ground are mainly contact and non-contact measurement^13–15^. The main types of contact ground height measurements are touchdown pressure sensors and imitation wheels, etc. Suggs et al.^16^ utilized the relationship between hydraulic pressure and depth of cut position to regulate the vertical position based on mechanical feedback control. However, the pressure of the hydraulic motor depends on soil conditions and crop density, which makes precise height control more difficult. Because the passive contact method usually lags behind changes in terrain, it is not a very desirable method for height measurement of crop divider. Wright et al.^17^ developed a mechanical/hydraulic system based on an articulated skid mounted in front of the base-cutter to monitor the vertical changes in the land. Zhao et al.^18^ designed a contact terrain sensing device suitable for use on a sugarcane harvester, and investigated the effects of device operating parameters on terrain height sensing performance. Xie et al.^19^ converted the changes in the feedback ground to cutting height as a signal generator for the head height control of the harvester. The use of pressure sensors has higher detection accuracy and better stability, but due to the conditions of sugarcane land such as soil type, soil moisture content and sugarcane planting density. It is necessary to carry out a baseline pressure test of the sugarcane land soil before each work, which is a complicated and time-consuming process. Measurement methods such as imitation wheels are direct and have less interference from the external environment. However, the inertia generated when the touching parts move up and down will cause inertial error affecting the measurement results, and at the same time, along with the long-term use of parts wear and tear of the accuracy of the situation. Commonly used non-contact measuring height ranging technology mainly includes ultrasonic ranging, image technology, microwave range, etc. Liu et al.^20^ proposed a video-based prediction system for combined harvester cutter height control. Huang et al.^21^ extracted key features by using image processing algorithms and employed an Otsu-based adaptive thresholding method to reconstruct the spatial coordinates of the feature points to guide the cutting height of sugarcane. However, the random presence of weeds and sugarcane leaves may interfere with the results of these methods. Zhang et al.^22^ implemented non-contact rubber trunk pruning surface profile measurement using laser ranging. Schembri et al.^23^ used ultrasonic sensors to detect the ground surface, but most of the signal was lost due to reflections from sugarcane leaves and weeds. Although methods based on ultrasonic sensors and image technology can be used to detect height in a variety of situations. Detection is made difficult by the fact that the sugarcane harvesting site is obscured by fallen sugarcane stalks and weeds, as well as the vibration and dust generated by the operation of the harvester. Using ultrasonic ranging and infrared ranging, infrared and ultrasound have limitations as they cannot penetrate sugarcane leaves and weeds. Therefore, the above methods are not well adapted to this complex scenario.

The millimeter-wave radar system is simple in structure, small in size, and has strong penetration capability. It has a short ranging time, small response time interval, and can realize real-time detection. Therefore, in this paper, millimeter wave radar is used for real-time detection of the height of the crop divider toes from the ground. And the parameters of the Kalman filtering method are adaptively adjusted to fit the data filtering for real-time measurements in this scenario. In this paper, we design a system to detect the height of crop divider toes from the ground, realize the real-time detection of the height and filter processing, and provide a reference for the design and development of an automatic height adjustment system for crop divider on sugarcane harvester in the future.

## Materials and methods

**Figure 1:**
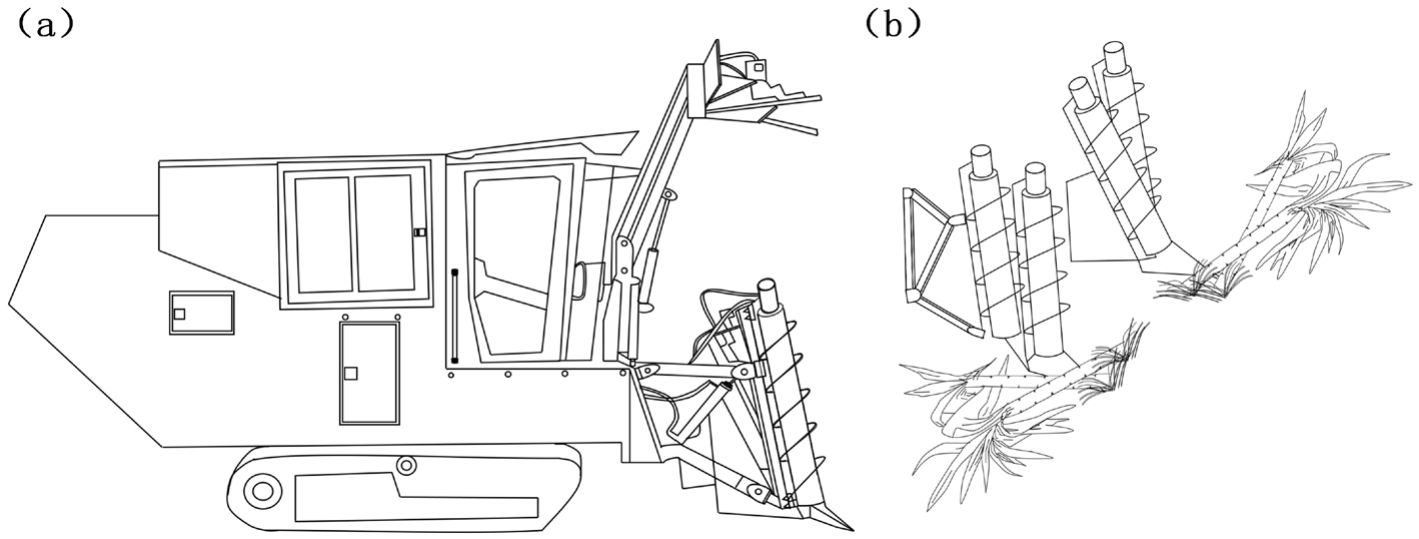
Schematic diagram of sugarcane harvester: (**a**) Crop divider position diagram; (**b**) Sugarcane lifting schematic diagram.

The crop divider is one of the critical devices on the sugarcane harvester, installed on both sides of the front end of the sugarcane harvester. Crop divider position diagram as shown in Fig. 1a, lifting sugarcane operation diagram as shown in Fig. 1b.

### Working principle

#### Millimeter wave ranging radar principle

Millimeter-wave radar ranges by transmitting a continuous wave with varying frequency in the sweep cycle, the echo reflected by the object has a specific frequency difference with the transmitted signal. The distance information between the target and the radar can be obtained by measuring the frequency difference^24^. There are two general ways of transmitting wave modulation: triangle wave and sawtooth wave modulation. Triangular wave modulation consists of two symmetrical linear FM continuous waves divided into upper and lower sweeps. Using the nature of the spectral symmetry of the upper and lower swept differential beat signals, the distance and speed are decoupled, and the distance to the target can be determined in one cycle using the differential beat Fourier approach. The way processing is simple and easy to implement. For sawtooth wave modulation, the Doppler shift and the frequency at relative rest cannot be solved in one cycle, and several cycles of continuous observation are needed for the solution. Therefore, the sensor module selected in this paper is HLK-LD303-24G ranging radar module, which adopts the triangular wave modulation method.

**Figure 2:**
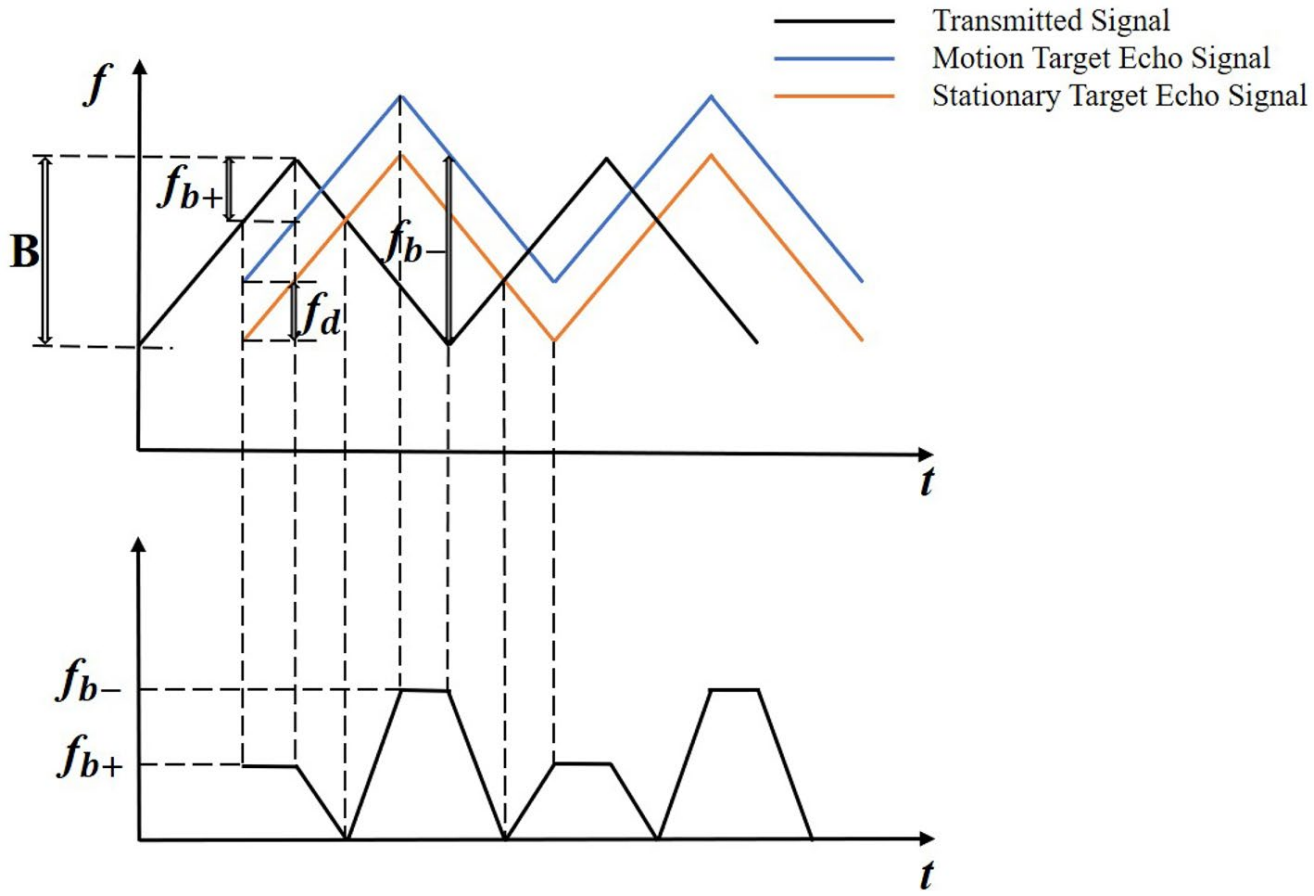
Principle diagram of millimeter wave distance measurement.

Figure 2 shows the principle diagram of millimeter wave ranging with triangular wave modulation. When the target is at rest, there is a time delay between the transmit signal and the return signal, and the time delay time is $\tau$ . Its value is $\tau =\frac{2D}{c}$, *D* is the distance of radar from the target object *c* is the propagation speed of the electromagnetic wave. The radar mixes the transmit signal with the echo signal to get the differential beat signal, and the differential beat signal gets the differential beat frequency through signal processing, and the frequency of the differential signal between the transmit signal and the echo signal is $f_b$ during the mixing process. When the target is in motion, due to the existence of the Doppler effect, the echo signal of the moving target will produce a Doppler shift $f_d$. At this time, the frequency of the output differential signal after mixing is shown in equations:

1 $$\begin{aligned} f_{b+}= & {} f_b-f_d \end{aligned}$$

2 $$\begin{aligned} f_{b-}= & {} f_b+f_d \end{aligned}$$

In Fig. 2, *B* denotes the FM bandwidth, $f_{b+}$ denotes the difference frequency at the rising edge of the triangular wave, $f_{b-}$ denotes the difference frequency at the falling edge of the triangular wave, and $f_d$ denotes the Doppler frequency shift. According to the principle, the following relationship exists for the distance *D* of the detected target.

3 $$\begin{aligned} D=\frac{c\tau }{2}=\frac{cT}{4B}f_b \end{aligned}$$

Combining equations (1), (2) and (3), the formula for the distance can be obtained as follows.

4 $$\begin{aligned} D=\frac{cT}{8B}\left( f_{b+}+f_{b-} \right) \end{aligned}$$

#### Kalman filtering

There are many interfering factors such as shaking and dust obscuration during the working process of the harvester, which will cause certain measurement errors and have a great impact on the accuracy of millimeter wave radar ranging. Therefore, it is necessary to filter the data measured by the range module to reduce the influence caused by the interference of the above factors and improve the accuracy of the final height data. The traditional data filtering method often needs to save multiple data before processing, which takes up a lot of memory, is not responsive enough, and has lagged. Kalman filtering only needs to store the parameters of the previous state and does not need to keep other historical data additionally, and the amount of operations is small, so it has the advantages of small memory occupation and fast calculation speed and can refresh and save data in real-time, so the Kalman filtering algorithm is chosen for filtering.

The Kalman filter mainly consists of two processes: time update and measurement update^25^. Time update mainly uses the best estimate of the last moment to derive the priori estimate of the current moment and the covariance matrix of the best estimate of the last moment from deriving the covariance matrix of the priori estimate of the current moment. Measurement update mainly combines the measured values of the current moment to correct the state and error covariance, and calculate the Kalman gain and the optimal estimate of the current moment.

The height *D* is taken as the state variable of this ranging system at the moment *k* . In this measurement process by default the heights of the two moments before and after do not change, which means that there is no switching of the state between the two neighboring moments, so the state transfer parameter *A* is set to 1. Since there is no control signal input in the system, the control signal $U_k$ is set to 0 and the control state parameter *C* is also set to 0. So the time update equation and the priori estimation error covariance $P_{k}^{-}$ at moment *k* are as follows.

5 $$\begin{aligned} D_{k}= & {} {\hat{D}}_{k-1}+W_{k} \end{aligned}$$

6 $$\begin{aligned} P_{k}^{-}= & {} P_{k-1}+Q \end{aligned}$$

where $W_k$ is the process error, $P_{k-1}$ is the error covariance at time $k-1$, and *Q* is the covariance of $W_k$ .

In the measurement update phase, the Kalman gain $K_k$ at moment *k* is first calculated based on the priori estimation error covariance $P_{k}^{-}$ obtained from the time update process and the covariance *R* of the measurement error $V_k$ .

7 $$\begin{aligned} K_{_k}=\frac{P_{k}^{-}}{P_{k}^{-}+R} \end{aligned}$$

Then, based on the Kalman gain $K_k$ and the state variables $D_k$ obtained in the prediction update phase, $Z_k$ is the measured height data, and the posterior state estimate ${\hat{D}}_{k}$ at moment *k* is calculated:

8 $$\begin{aligned} {\hat{D}}_{k}=D_k+K_k\left( Z_k-D_k\right) \end{aligned}$$

Finally, the error covariance $P_k$ at moment *k* is updated based on the priori estimation error covariance $P_{k}^{-}$ and the Kalman gain $K_k$ :

9 $$\begin{aligned} P_k=\left( I-K_k \right) P_{k}^{-} \end{aligned}$$

where: *I* is the unit matrix.

The above is the basic process of the Kalman filtering algorithm, by setting initial values ${\hat{D}}_{0}$ and $P_0$ for the state vector and covariance matrix, the optimal estimation of the current state can be obtained by constantly updating according to the measured values.

#### Data pre-processing and Kalman adaptive adjustment

Due to the influence of disturbing factors in the measurement process, the chance errors caused by interference were removed by pre-processing the data. The chance error is judged by Eq. (10):

10 $$\begin{aligned}{} & {} \left| a_k-a_{k-1} \right| \ge 5 \end{aligned}$$

11 $$\begin{aligned}{} & {} a_k=\frac{a_{k-1}+a_{k-2}}{2} \end{aligned}$$

where $a_k$, $a_{k-1}$ and $a_{k-2}$ denote the data input to the module at the moments *k*, $k-1$ and $k-2$.According to the datasheet description of the selected radar module, its detection accuracy is 5 cm, and the allowable error threshold is set to 5. When the absolute value of the error between the current moment and the previous moment is greater than or equal to 5, it is considered to be a chance error. Replace it with Eq. (11), which means replacing it with the average of the previous two moments of data. The pre-processed data is used as the input data at the current moment and then Kalman filtering is utilized to obtain the predicted values. The predicted and observed values are corrected by Kalman filter gain coefficients to obtain the final height data. When the height actually changes, judgment and replacement of the above-mentioned accidental errors are not performed.

Considering the actual situation of the sugarcane field, where there is a height variation in the unevenness of the sugarcane field. In the recursive process of the Kalman filter algorithm, the error covariance $P_k$ and Kalman gain $K_k$ decay rapidly to fixed values. It will make the newly measured data less corrective to the state variables and the processed data will be slowly recursive to near the true value. By designing a judgment that the height has changed. When it is judged that the actual height has changed, the Kalman filtering algorithm is adjusted. To maintain the ability of the new measurement data to correct for state variables, which in turn allows the height measurement system to obtain a faster response estimate.

**Figure 3:**
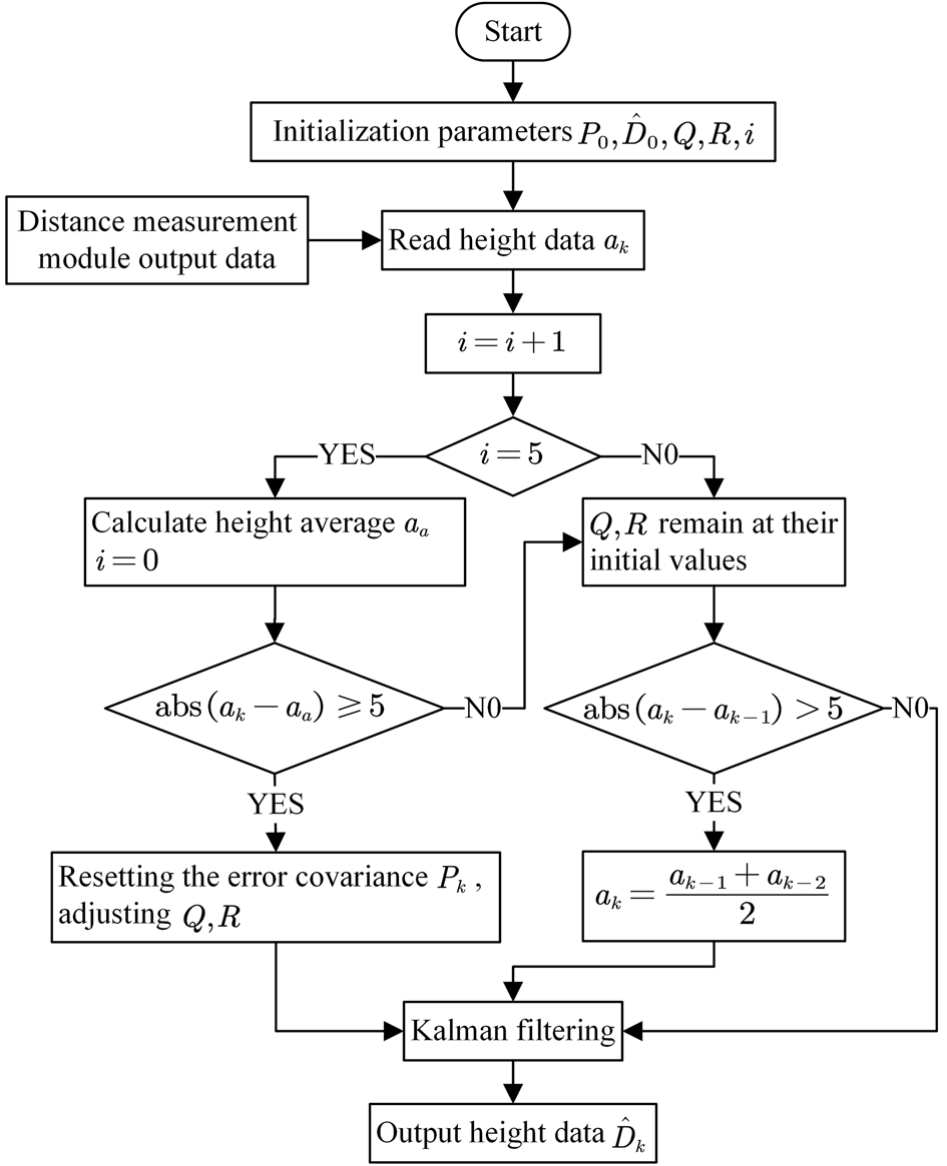
Filtering algorithm process.

The measured value should be trusted more when the height changes. From Eq. (8) it is known that the Kalman gain $K_k$ should be increased, and from Eqs. (6) and (7) it can be seen that the smaller the *R* the larger the $K_k$ , and the larger the *Q* the larger the $K_k$. By adjusting the values of *Q* and *R*, the system gives more confidence in the measured values and responds more quickly to changes. Design a threshold value *i* , which indicates the time when the height change is judged. If it is too small, accidental errors will be mixed into it and affect its judgment result; if it is too large, it will make the response time of the system too long. Therefore, *i* takes 0.5 seconds of sampling time, which is 5 sampling points. Set a counter with the threshold *i* as the upper limit of the counter. When the counter reaches the upper limit, the counter is cleared to zero and the height data entered by the module within the *i* sampling point is averaged. Compare the height data entered at this moment with the average value. If the difference is greater than 5, determine that the height has changed at this point. The Kalman filtering algorithm is then adjusted, which is to reset the error covariance and adjust the values of *Q* and *R*. Then the Kalman filtering operation is performed to calculate and output the height value at the current moment. If the difference is less than or equal to 5, it is judged that there is no significant change in height at this time. The specific algorithmic process is shown in Fig. 3.

### Simulated test program

#### Test platform construction

**Figure 4:**
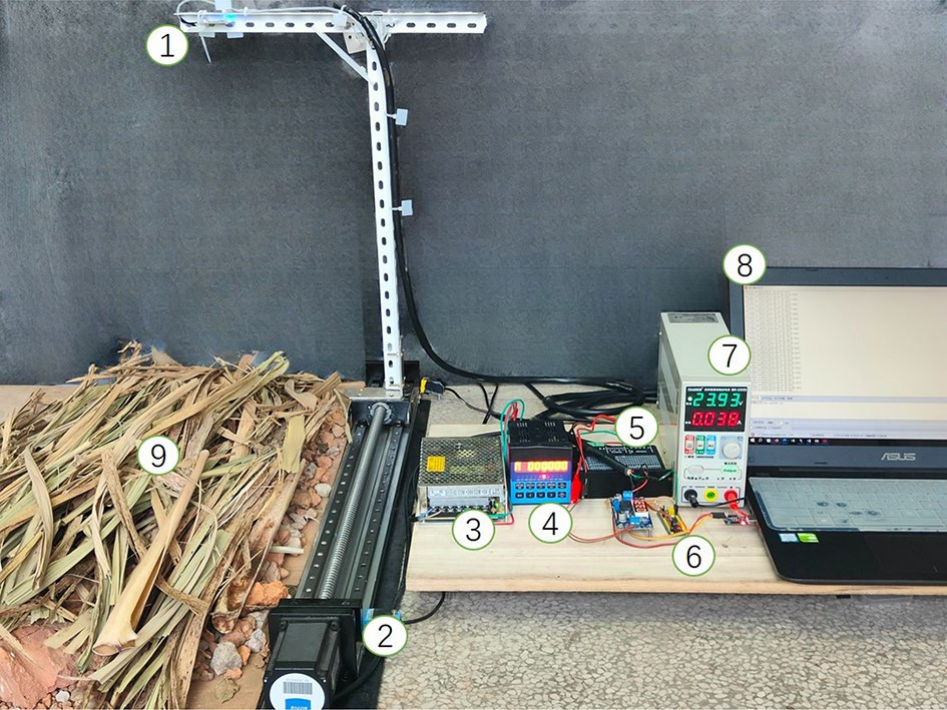
Simulation test platform: (1) Millimeter wave radar ranging module, (2) Slider module, (3) DC power, (4) Stepper motor controller, (5) Stepper motor driver, (6) STM32 module, (7) AC power, (8) Data acquisition, (9) Simulated sugarcane fields (soil covered with sugarcane leaves).

To test the performance of a millimeter wave radar range module-based method for detecting the height of the crop divider from the ground in sugarcane harvester. Considering the reality of harvesting situations there will be harvesters traveling at different speeds and sugarcane fields will be covered with sugarcane leaves, weeds, and so on. A platform for simulation testing of height measurement with an adjustable forward speed function was built for testing. The test platform, as shown in Fig. 4. The platform mainly includes the slide table unit and the millimeter wave radar sensing detection unit. The slide table unit mainly includes a stepper motor, driver, controller, etc. The running speed and the distance moved by the slider are set by the controller to realize the simulation of the sugarcane harvester moving scene. The millimeter wave radar sensing detection unit mainly includes measurement hardware (HLK-LD303-24G ranging radar module), height detection data processing system (MCU STM32F103C8T6), and power supply module, and the height detection processing system realizes the fast saving and processing of millimeter wave detection data. The millimeter-wave radar ranging module used in the experiment has a transmitting frequency of 24 to 24.25 GHz, an operating voltage of DC5V, and a detection blind spot of 10 cm.

#### Simulated test design

Since the sugarcane harvester will have different driving speeds and raise a lot of dust during the working process, the sugarcane field will also be covered with peeled sugarcane leaves and weeds, which all bring significant interference to the range accuracy. Therefore, the forward speed of the harvester and the thickness of the ground vegetation cover were selected as the test factors, and the detection height was taken as the evaluation index. To detect the influence of the forward speed and the vegetation cover thickness on the accuracy of the range, Two sets of one-factor variation tests were designed on the simulated test platform: the forward speed variation test and the vegetation cover thickness test, and two-factor test was also designed. The sliding speed of the slider was adjusted to 0.08 m/s, 0.10 m/s, and 0.12 m/s by stepping motor to simulate the change of forward speed of the sugarcane harvester; and the ground vegetation cover was changed to no cover, 3 cm, and 6 cm to simulate the situation of sugarcane leaves and weeds cover on the sugarcane field. The factor settings are shown in Table 1, and a one-factor variation test and a two-factor test were conducted for these two cases to verify their effects on ranging accuracy.

**Table 1:** Factor settings.

Factors	Levels		
	1	2	3
Forward speed(m/s)	0.08	0.1	0.12
Vegetation cover thickness(cm)	0	3	6

In the case where external factors have an influence on the ranging, a fixed-height test was designed in order to compare and analyze the errors of the original measured data and the filtered processed data with the actual data , respectively. For this purpose, the forward speed of the sugarcane harvester and the ground cover thickness were selected as the test factors, and the error between the detected height and the actual height was used as the evaluation index for a one-factor variation repetition test. The actual height of the sensor was fixed at 50 cm from the ground, and the three forward speeds were set as 0.08 m/s, 0.10 m/s, and 0.12 m/s, and the three vegetation cover thicknesses were no cover, 3 cm, and 6 cm, respectively. The forward speed and ground vegetation cover thickness were varied sequentially on a simulated test platform, and seven replicate experiments were conducted for each combination. Record the original data measured by the ranging module and the filtered processed data for each experiment.

Due to the inequality of sugarcane harvesting field, in order to simulate the real application situation, an experiment was designed to test the change of the installation position of the ranging module in terms of the height from the ground. By varying the thickness of the ground soil, this simulates changing the actual height of the ranging module from the ground. The total length of the sliding table was divided into three equal sections and different soil thicknesses were set for the ground in these three sections, with soil thicknesses in 10 cm increments, which means that the actual heights of the installation locations above the ground were 50 cm, 40 cm, and 30 cm, respectively. The thickness of the ground vegetation cover was fixed at 3 cm, and the forward speed was set at 0.10 m/s.

### Field test program

**Figure 5:**
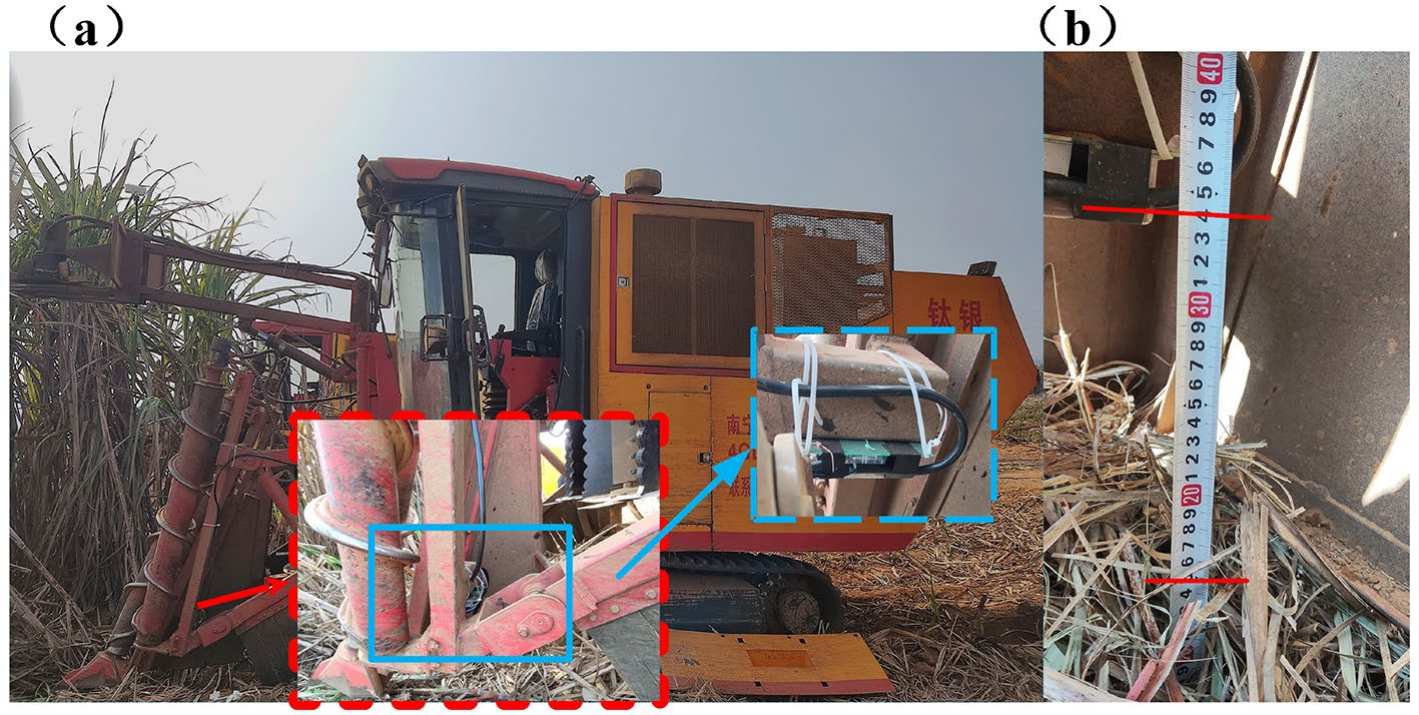
Field test situation map: (**a**) Distance measurement module installation location; (**b**) Actual height of the installation position from the ground 34 cm and vegetation cover thickness of sugarcane field 5 cm.

Experiment on the height measurement of the crop divider toes from the ground at the sugarcane harvesting site. The experiment was conducted at the sugarcane planting base of Taoxu Town, Heng County, Hengzhou City, Guangxi Zhuang Autonomous Region, China. The millimeter wave radar distance measurement module was mounted on the rear end of the baffle plate of the crop divider of the sugarcane harvester. Since the transceiver shares the antenna, the transceiver transfer switch does not receive signals during the transmitter operation, and the reflected target echoes cannot be received during this period, so the sensor has a measurement blind spot. If the installation position is too low, the distance cannot be measured if it is in the blind area, and it is easily damaged by scraping with the ground, so the distance measuring sensor module is installed to a suitable position on the crop divider. The millimeter wave radar ranging module installation position is shown in Fig. 5a. Measure the distance from the ground to the installation position. The height of the installation position from the crop divider toes is fixed. The measured distance minus the fixed height of the installation position from the crop divider toes is the height of the crop divider toes from the ground.

Manually measure the height of the mounting position from the ground as the true height. As shown in Fig. 5b, the height of the mounting position is 34 cm and the vegetation cover thickness of the sugarcane field is 5 cm. Six groups of experiments for height detection were performed during the harvesting work. Experiments are conducted to verify whether the range of measurement of the module is reasonable and whether the accuracy meets the requirements after real-time data processing.

## Results and discussion

### Simulated test results

Simulation tests were conducted to analyze the effects of forward speed and vegetation cover thickness on the ranging system, and to analyze the effect of height from the ground on the filtering effect and response time of the system.

#### External factors influence the test

As described in the simulated test program, forward speed as a factor for the one-factor variation test, set three-speed levels were 0.08 m/s, 0.10 m/s, and 0.12 m/s. Vegetation cover thickness was taken as 3 cm, the height of the fixed sensor from the ground was 50cm, each speed was repeated seven times of the test, the corresponding height data results were recorded, and the mean square error of the data was calculated, One-way Analysis of Variance (ANOVA) was performed on the test results of changing the forward speed. The results are shown in Table 2.

**Table 2:** The results of one-way ANOVA of forward speed.

Source	SS	DF	MS	F	P
Forward speed	4.527	2	2.263	14.52	0.0002
Error	2.807	18	0.156		
Total	7.334	20			

The vegetation cover thickness was used as a factor in the one-factor variation test, and three vegetation cover levels were set as no cover, 3 cm, and 6 cm. The forward speed was 0.10 m/s, and the height of the fixed sensor from the ground was 50 cm. Each speed was repeated seven times, the corresponding height data results were recorded, and the mean square error of the data was calculated, One-way ANOVA was performed on the test results of changing the vegetation cover thickness. The results are shown in Table 3.

**Table 3:** The results of one-way ANOVA of vegetation cover thickness.

Source	SS	DF	MS	F	P
Vegetation cover thickness	5.904	2	2.952	12.44	0.0004
Error	4.271	18	0.237		
Total	10.175	20			

In Tables 2 and 3, Source denotes the source of variance, SS denotes the sum of squares, and DF denotes the degrees of freedom for each source. If the factor has three levels, the degrees of freedom are 2. MS indicates mean square, which is the mean square of the sum of squares divided by the degrees of freedom. F indicates the F-ratio, which is calculated by dividing the MS of the factor by the MS of the error. The *P* value is used to determine if a factor is significant, usually compared to a value of 0.05. If the *P* value is less than the 0.05 value, the factor is significant. For the measured error, the *P* value was 0.0002 (P<0.05) for forward speed and 0.0004 (P<0.05) for vegetation cover thickness, which indicated that forward speed and vegetation cover thickness have a significant effect on range accuracy.

**Table 4:** The results of two-way ANOVA for forward speed and vegetation cover thickness.

Source	SS	DF	MS	F	P
Forward speed	6.925	2	3.462	27.66	0.0003
Vegetation cover thickness	5.873	2	2.936	18.42	0.0004
Forward speed $\times$ Vegetation cover thickness	1.073	4	0.2682	1.787	0.2028
Error	3.602	24	0.1501		
Total	17.473	32			

The results of the two-way ANOVA are shown in Table 4. The *P* values of the forward speed and vegetation cover thickness were both less than 0.001, which indicated that each of these two factors had a significant effect on the ranging accuracy. The *P* value under the interaction of the forward speed and vegetation cover thickness was higher than 0.05, which indicated that the interaction of these two factors did not have a significant effect on the ranging accuracy. Consequently, the main effect of this study was significant, while the interaction effect was not significant. Because the main effects of the forward speed and vegetation cover thickness were independent of each other, the interaction of the two factors did not affect the ranging accuracy.

#### Fixed height test

**Figure 6:**
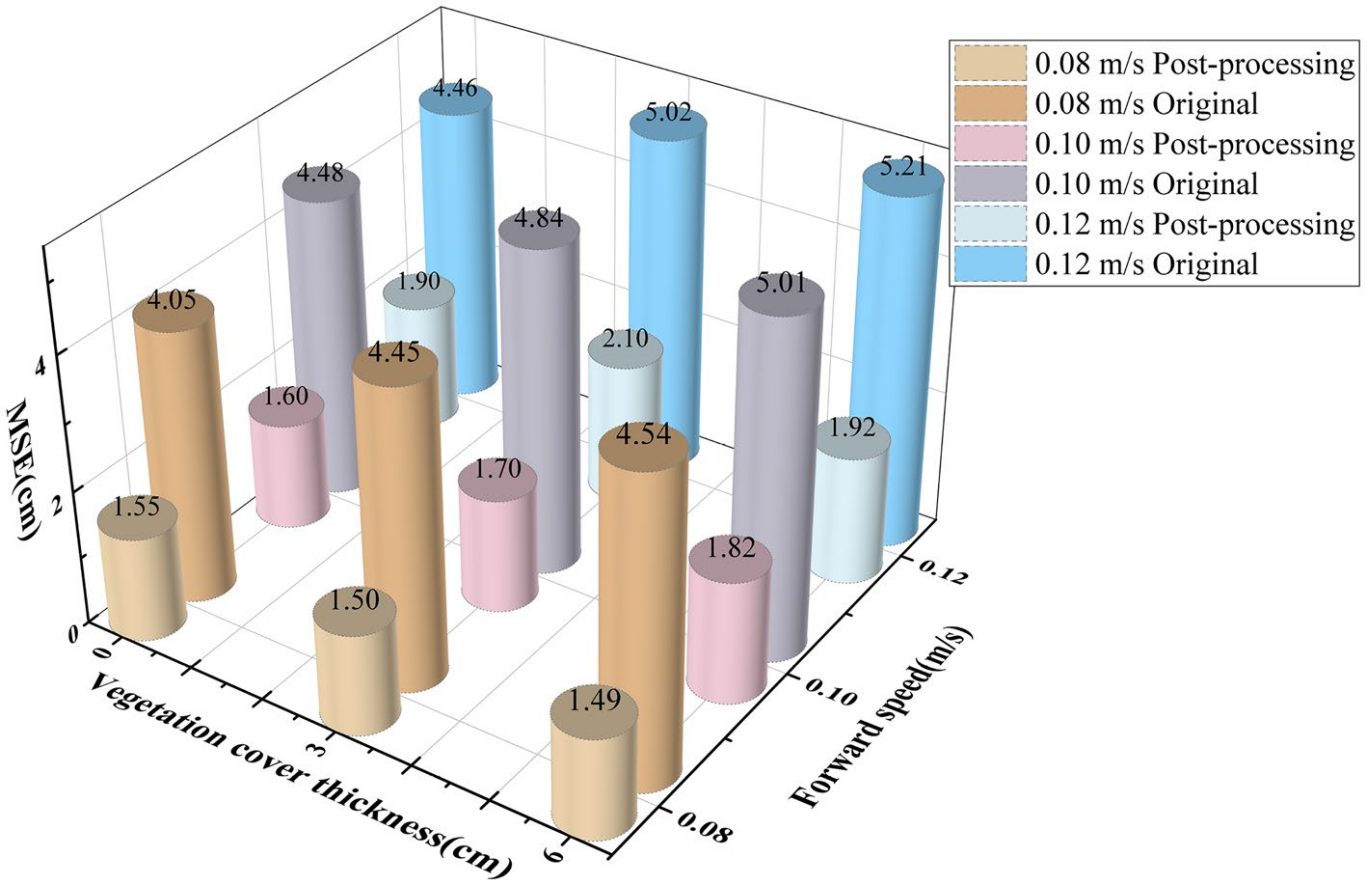
Constant height from ground error analysis diagram.

The above ANOVA of the effects of forward speed and vegetation cover thickness on the ranging accuracy of the millimeter wave radar shows that both factors have a significant effect on the ranging accuracy of this ranging module. Fixed-height tests were conducted on the simulated test platform with two-by-two combinations of each of three levels of forward speed and ground vegetation cover thickness, with seven experiments repeated for each combination. Based on recording the original data measured by the ranging module for each test and the filtered processed data, respectively, they were compared with the actual height and the mean square error was calculated. The errors obtained were averaged and the test results are shown in Fig. 6.

From the data analysis in Fig. 6, it can be seen that the error of the measured height data is more extensive as the vegetation cover thickness increases; the error of the measured height data gradually increases as the forward speed gradually increases. Before filtering, the maximum mean value of error is 5.21 cm, which occurs when the vegetation cover thickness is 6 cm, the forward speed is 0.12 m/s, and the minimum mean value of error is 4.05 cm, which occurs when there is no vegetation cover thickness and the forward speed is 0.08 m/s. After data filtering, the mean value of error is reduced, and the maximum mean value of error is 2.10 cm, which occurs when the vegetation cover thickness is 3 cm and the forward speed is 0.12 m/s, and the minimum mean value of error is 1.49 cm, which occurs when the vegetation cover thickness is 6 cm, and the forward speed is 0.08 m/s.

#### Height change test

**Figure 7:**
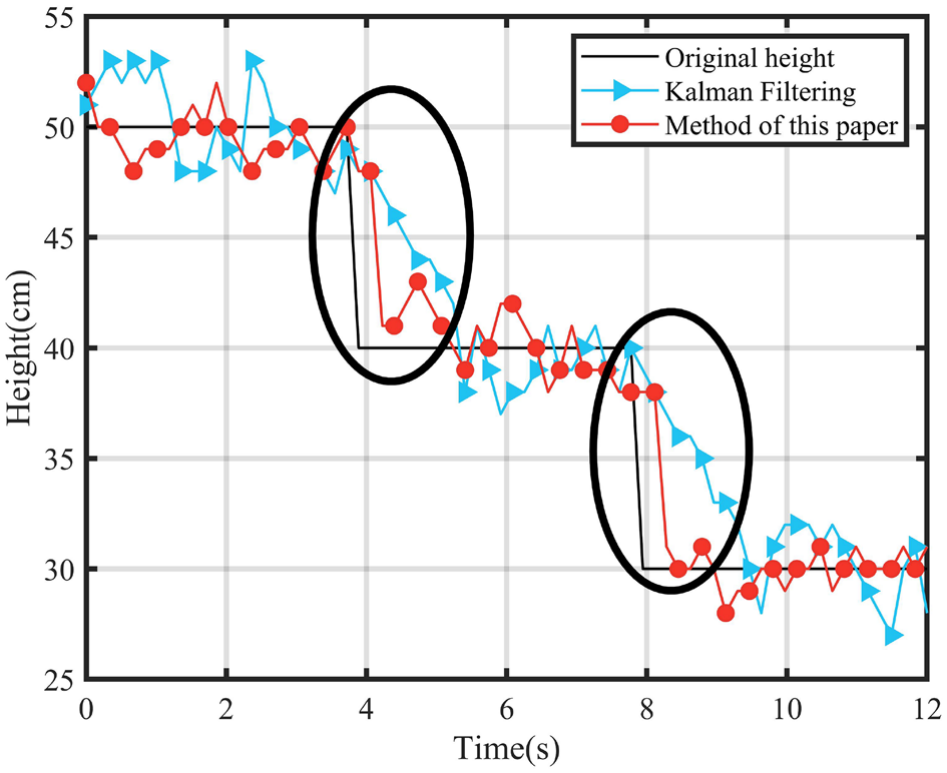
Comparison of Kalman filtering and the method in this paper.

Simulated tests of height changes were carried out, and the original data obtained from the ranging module were processed using the traditional Kalman filtering method and the method of this paper, respectively. The results are shown in Fig. 7, which shows the actual heights at different times, the heights after the Kalman filtering process and the heights after the method in this paper. When the measured height changes, the two places circled in the figure, the method in this paper can respond faster to the actual height neighborhood.

### Field trial validation

**Figure 8:**
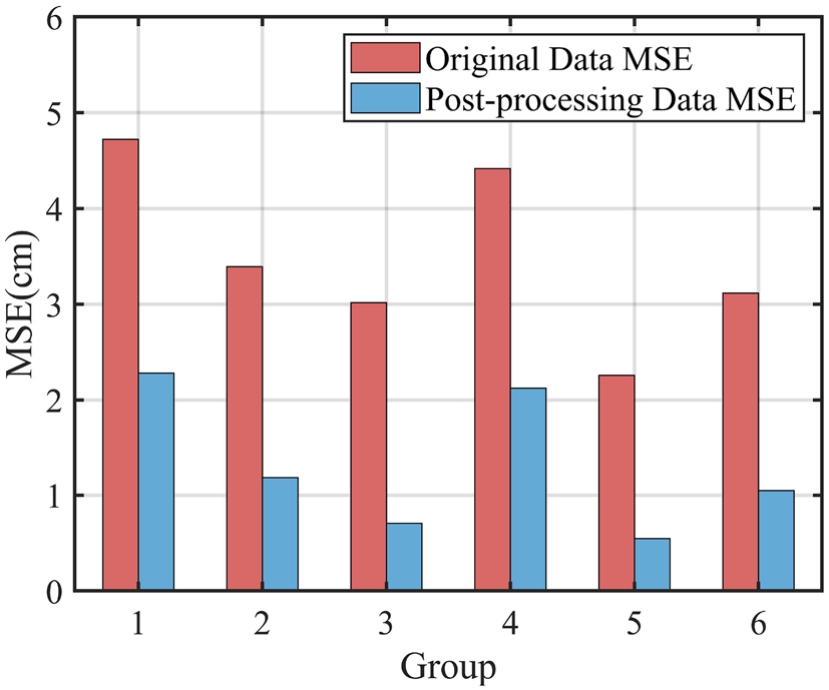
Mean square error of the original data and the data processed by the method in this paper.

In order to verify the feasibility of this ranging system, height measurement tests were conducted in the field. The ranging module was tested by mounting it on the rear end of the baffle of the crop divider of a sugarcane harvester, as described in the field test program. Manual and systematic height measurements were recorded. A total of six sets of experiments were conducted during the operation of the sugarcane harvester, with 20 manual height measurements in each set of experiments. The actual height data measured manually were compared and analyzed with the original data measured by the system and the filtered processed data. The mean square error calculations were carried out respectively and the experimental results are shown in Fig. 8.

**Figure 9:**
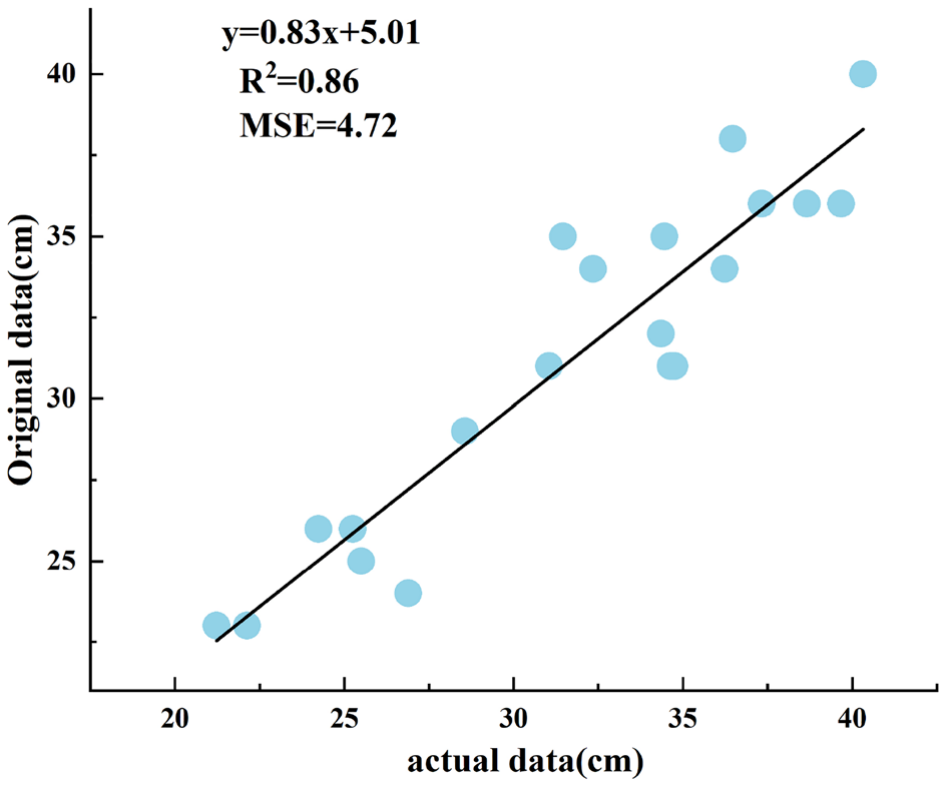
Correlation of original data.

**Figure 10:**
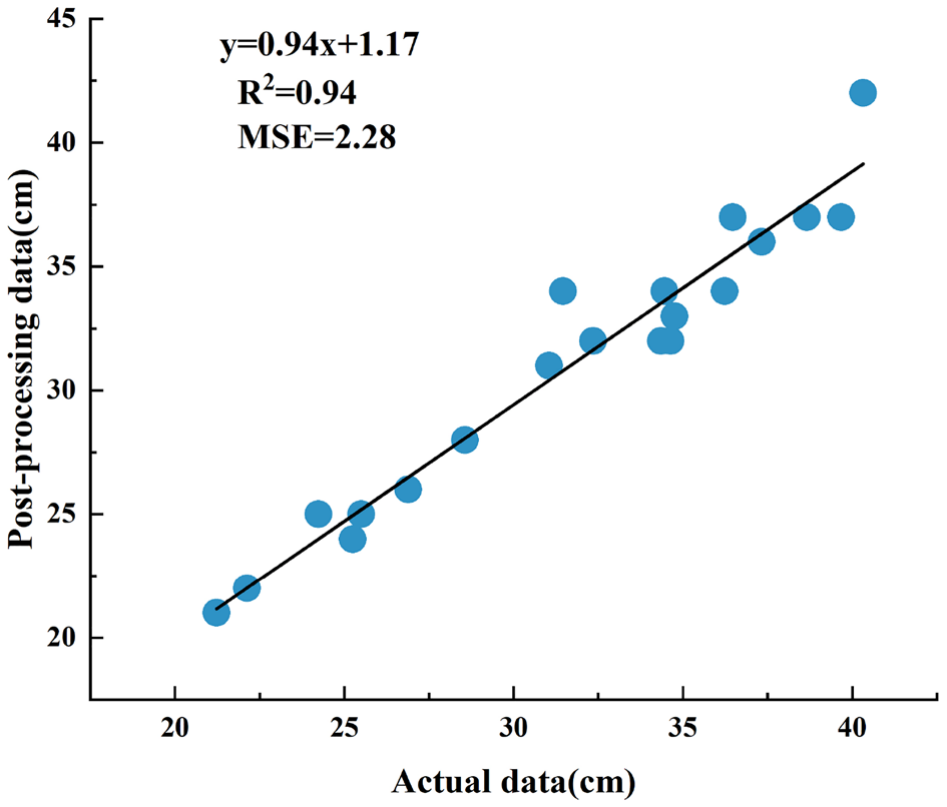
Correlation of the processed data.

As can be seen from Fig. 8, the minimum error after the filtering process is 0.55 cm and the maximum error is 2.28 cm. The correlation between the manually measured data and the systematically measured original and processed data for the first set of experiments is shown in Figs. 9 and 10. The correlation between the original height measured by the system and the manually measured height $R_{}^{2}$ = 0.86, and the correlation between the height processed by the system and the manually measured height $R_{}^{2}$ = 0.94. A higher correlation indicates greater accuracy. The results show that the ranging system can perform height measurements in real complex environments. Due to the influence of various factors in the environment at the time of measurement, such as different thicknesses of vegetation cover, etc., there are different error situations in the measurement results. However, after the data filtering process in this paper, the errors can be reduced, and the effect of improving the measurement accuracy was achieved.

The methods in this paper are different from those mentioned in the reference section of this paper to improve the performance of crop divider by way of optimizing the structure of the crop divider. For applications in sugarcane fields where the terrain is uneven, requiring the operator to manually adjust the height of the crop divider and where it is difficult to accurately observe the height by sight. The millimeter wave radar ranging module used in this paper can penetrate a certain amount of sugarcane leaf cover, which is advantageous for the complex environment in the field.In addition, height data were measured and filtered in real time during the operation of the harvester. According to the test results can be seen in the data error is in the acceptable range of 2.5 cm, and data processing response time is faster, the hardware cost is low, the height of the measurement effect and cost advantage is obvious. However, this method has an impact on the measurement performance due to only testing the effects of forward speed and vegetation cover thickness, but in real production situations, the sugarcane harvester’s violent vibration, huge dust, etc. can have an impact on the measurement performance. The method was not integrated with the height control of the crop divider. Therefore, future research needs to integrate the method with the lifting and lowering control of crop divider in order to facilitate the implementation of the method in a practical setting as soon as possible.

### Plant material collection and use permission

The sugarcane materials collected in this study have been approved by the owner of the sugarcane planting base of Taoxu Town, Heng County, Hengzhou City, Guangxi Zhuang Autonomous Region, China. The collected sugarcane materials and research activities are in line with the laws and regulations of the Guangxi Zhuang Autonomous Region, China. Authors complied with the IUCN Policy Statement on Research Involving Species at Risk of Extinction and the Convention on the Trade in Endangered Species of Wild Fauna and Flora for the collection of plant or seed specimens. Authors declare that no wild plants were collected and/or used in this scientific work.

### Ethics approval and consent to participate

We all declare that manuscript reporting studies do not involve any human participants, human data, or human tissue. So, it is not applicable.

### Complies with international, national and/or institutional guidelines

This study complies with relevant institutional, national, and international guidelines.

## Conclusion

Crop divider is an important device on the sugarcane harvester, if it can always move against the ground during the forward process, it can lift more fallen sugarcane, improve the rate of sugarcane harvesting, and increase the economic benefits of sugarcane farmers. The height detection of the crop divider toes is the basis for precise control of the crop divider toes against the ground, this paper designed a height detection system for measuring the height of the crop divider toes from the ground. Due to the complexity of the working environment in the field, in order to improve the measurement accuracy, the millimeter-wave radar ranging module was used in the height data acquisition part of the system and filtered. A Kalman adaptive adjustment filtering method was proposed to address the problem of slow filtering response.

After the tests were conducted on the simulated test platform, the ANOVA concluded that the forward speed of the sugarcane harvester and the vegetation cover thickness on the ground had a significant effect on the measurement accuracy, and the interaction between the forward speed and the vegetation cover thickness did not have a significant effect on the ranging accuracy. By designing simulation tests for three forward speeds and three vegetation cover thicknesses with constant measurement height, the filtering method proved to be effective in improving the accuracy, with error values less than 2.5 cm. Tests were designed to measure changes in height, and the Kalman filter was readjusted to enable a fast response to changes in height near the actual value by judging the change in height. Considering the complexity of the actual field application environment, the test was validated in the field environment and the error range of the processed data was 0.55cm-2.28cm. The correlation also improved when compared to manually measured data. The results show that the ranging system can be applied to the real environment. Therefore, this system can provide a reference for the future design of automatic lifting and lowering of crop divider on sugarcane harvesters.

## Data Availability

The datas used and/or analysed during the current study available from the corresponding author on reasonable request.
